# Amorfrutin B Protects Mouse Brain Neurons from Hypoxia/Ischemia by Inhibiting Apoptosis and Autophagy Processes Through Gene Methylation- and miRNA-Dependent Regulation

**DOI:** 10.1007/s12035-022-03087-9

**Published:** 2022-11-03

**Authors:** Karolina Przepiórska, Agnieszka Wnuk, Cordian Beyer, Małgorzata Kajta

**Affiliations:** 1 Maj Institute of Pharmacology, Polish Academy of Sciences, Department of Pharmacology, Laboratory of Neuropharmacology and Epigenetics, Smetna Street 12, 31–343 Krakow, Poland; 2 Institute of Neuroanatomy, Medical Faculty, RWTH Aachen University, 52074 Aachen, Germany

**Keywords:** Hypoxia, Ischemia, Perinatal asphyxia, Stroke, PPARγ, Autophagy

## Abstract

Amorfrutin B is a selective modulator of the PPARγ receptor, which has recently been identified as an effective neuroprotective compound that protects brain neurons from hypoxic and ischemic damage. Our study demonstrated for the first time that a 6-h delayed post-treatment with amorfrutin B prevented hypoxia/ischemia-induced neuronal apoptosis in terms of the loss of mitochondrial membrane potential, heterochromatin foci formation, and expression of specific genes and proteins. The expression of all studied apoptosis-related factors was decreased in response to amorfrutin B, both during hypoxia and ischemia, except for the expression of anti-apoptotic BCL2, which was increased. After post-treatment with amorfrutin B, the methylation rate of the pro-apoptotic *Bax* gene was inversely correlated with the protein level, which explained the decrease in the BAX/BCL2 ratio as a result of *Bax* hypermethylation. The mechanisms of the protective action of amorfrutin B also involved the inhibition of autophagy, as evidenced by diminished autophagolysosome formation and the loss of neuroprotective properties of amorfrutin B after the silencing of *Becn1* and/or *Atg7*. Although post-treatment with amorfrutin B reduced the expression levels of *Becn1*, *Nup62*, and *Ambra1* during hypoxia, it stimulated *Atg5* and the protein levels of MAP1LC3B and AMBRA1 during ischemia, supporting the ambiguous role of autophagy in the development of brain pathologies. Furthermore, amorfrutin B affected the expression levels of apoptosis-focused and autophagy-related miRNAs, and many of these miRNAs were oppositely regulated by amorfrutin B and hypoxia/ischemia. The results strongly support the position of amorfrutin B among the most promising anti-stroke and wide-window therapeutics.

**Supplementary Information:**

The online version contains supplementary material available at 10.1007/s12035-022-03087-9.

## Introduction

Stroke is the most common cerebrovascular disease, and approximately 85% of stroke cases are of ischemic origin, resulting from vessel occlusion and impaired blood flow to the brain. Data suggest that 1/3 of patients with stroke die, pointing to the ineffectiveness of current therapies. In addition to stroke, perinatal asphyxia is another neurological disorder that leads to the death of 4 million newborns per year globally [1]. The dysregulation of gas exchange leads to metabolic acidosis of organs, including the brain, and results in ischemic encephalopathy [2]. Current therapies for ischemic stroke and perinatal asphyxia are recombinant tissue plasminogen activator (rtPA) and hypothermia; however, these therapies have several limitations, including a narrow therapeutic window (up to 6 h) and other adverse effects [3, 4]. For this reason, patients are often not admitted to treatment, which prompts the search for new therapies against stroke and perinatal asphyxia.

Disruption of blood and oxygen supply to the brain leads to a cascade of pathological responses and subsequent cell death. The main mechanism in hypoxic and hypoxic-ischemic brain injury is excitotoxicity, resulting from excessive accumulation of calcium and membrane depolarization [5]. It sequentially leads to dysfunction of mitochondria and apoptosis, a form of programmed cell death characterized by DNA fragmentation, apoptosis-associated heterochromatin foci and apoptotic body formation. An intermediary chromatin remodeling state that relies on formation of apoptosis-associated heterochromatin foci finally leads to apoptotic body formation [6, 7]. It is highly connected with autophagy (“self-eating”), which may have protective involvement or pro-apoptotic favor under ischemic or hypoxic conditions. Although some studies propose protective involvement of autophagy in cerebral ischemia, others suggest that autophagy may switch the neuronal injury to apoptosis and cell death under ischemic or hypoxic conditions [8, 9]. Apoptosis and autophagy can be regulated *via* miRNAs, small noncoding RNAs that downregulate gene expression by mRNA cleavage or translational repression [10]. Recent studies have shown that the differential expression of miRNAs (e.g., *miR-124* and *miR-181b*) in the brain has a pivotal role in the pathological mechanism of stroke and cerebral asphyxia, and the potential use of miRNA antagonists may be a promising direction for the improvement of pharmacotherapy [11, 12].

Peroxisome proliferator–activated receptor gamma (PPARγ) is a ligand-activated transcription factor that functions to regulate insulin sensitivity, glucose and lipid metabolism, as well as cell proliferation and inflammation [13, 14]. Due to its ability to adjust many key metabolic processes, it is considered a promising pharmacological target in chronic neurological conditions. The compounds investigated in the context of neuroprotection are thiazolidinediones (TZDs), PPARγ full agonists, which were originally found to have applications as antidiabetic drugs. TZDs such as pioglitazone and rosiglitazone improve the survival of neurons and glial cells during ischemic damage by reducing inflammation and exhibiting antiatherosclerotic properties [15]. Unfortunately, recent research reports about the hepatotoxicity and cardiotoxicity of TZDs resulting in partial withdrawal of these compounds from the pharmaceutical market [16, 17].

Amorfrutin B isolated from *Amorpha fruticosa* is a selective PPARγ modulator (SPPARγM) that may have a safer pharmacological profile than TZDs. In comparison to full agonists, SPPARγMs activate the receptor in a specific way by modulating the interaction of PPARγ with transcriptional activators or repressors [18]. Recent studies have indicated that the application of amorfrutin B may constitute a promising approach to treat insulin resistance, type 2 diabetes, or metabolic liver diseases [19–21]. Importantly, in diabetes and diet-induced obese mouse models, the compound did not show side effects of TZDs, such as osteoblastogenesis, weight gain, and fluid retention [21]. Among many compounds, amorfrutin B has the lowest binding affinity constant (*K*_*i*_) to purified PPARγ, which makes this compound the most selective and potent SPPARγM [22]. Moreover, amorfrutin B is a highly lipophilic molecule that crosses the blood–brain barrier (BBB) and accumulates in brain tissue, which is a desirable feature of potential drugs directed against diseases of the central nervous system [23]. Despite the aforementioned studies, little is known about the potential of amorfrutin B in the treatment of neural degeneration caused by hypoxia and ischemia. Our recent study showed that amorfrutin B, administered even 6 h after injury, evokes a strong neuroprotective effect against hypoxia and ischemia by increasing the viability of neuronal cells and mitochondrial integrity as well as reducing oxidative stress and the accompanying DNA damage. The neuroprotective effect of amorfrutin B involves PPARγ activation and epigenetic modifications, which position this compound among the most promising anti-stroke therapeutics [24].

Due to the diverse etiologies and courses of hypoxic and ischemic injuries, it is crucial to achieve neuroprotective effects against various mechanisms accompanying neurodegeneration. For this reason, the current research is the first to check the effectiveness of amorfrutin B in relation to apoptosis and autophagy. Moreover, the research includes the analysis of the epigenetic status of neuronal cells specifying the miRNA profile. Multidirectional analysis of the action of amorfrutin B would help to define the features of this potential drug that possesses neuroprotective capacity against hypoxia and ischemia within a wide therapeutic window.

## Methods

### Materials

The JC-10 Mitochondrial Membrane Potential Assay Kit, Z-IETD-FMK, Z-LEHD-FMK, TDZD 8, and SB 203580 were purchased from Abcam (Cambridge, Great Britain). A hypoxia modular incubator chamber was obtained from Billups-Rothenberg, Inc. (San Diego, CA, USA). Phosphate-buffered saline (PBS) was purchased from BIOMED LUBLIN (Lublin, Poland). The CYTO-ID**®** Autophagy detection kit was from Enzo Life Sciences, Inc. (Farmingdale, NY, USA). B27 and neurobasal media were obtained from Gibco (Grand Island, NY, USA). The oxygen analyzer was from Greisinger (Regenstauf, Germany). Bradford reagent was obtained from Bio–Rad Laboratories (Munchen, Germany). The cytotoxicity detection kit was purchased from Roche Diagnostics GmbH (Mannheim, Germany). ELISA kits for FAS, FASL, BAX, BCL2, GSK3β, BECN1, ATG7, and MAP1LC3B were purchased from Bioassay Technology Laboratory (Shanghai, China). An ELISA kit for AMBRA1 was purchased from ELK Biotechnology (Wuhan, China). The culture plates were obtained from TPP Techno Plastic Products AG (Trasadingen, Switzerland). The *Becn1* and *Atg7* siRNAs were purchased from Santa Cruz Biotechnology, Inc. (Santa Cruz, CA, USA). Amorfrutin B, Z-DQMD-FMK, SP600125, temsirolimus, SBI-0206965, spautin-1, MRT68921, *L*-glutamine, fetal bovine serum (FBS), dimethyl sulfoxide (DMSO), radioimmunoprecipitation assay buffer (RIPA) buffer, protease inhibitor cocktail for mammalian tissues, penicillin–streptomycin antibiotics, and poly-*L*-ornithine hydrobromide were obtained from Sigma-Aldrich (St. Louis, MO, USA). AllStars Negative Control siRNA, the RNeasy Mini Kit, the EpiTect MethyLight PCR kit, the EpiTect Bisulfite kit, and the miRNeasy Mini kit were obtained from Qiagen (Hilden, Germany). INTERFERin was obtained from PolyPlus Transfection (Illkirch, France). Sets of TaqMan probes (fully methylated and fully unmethylated probes for the *Bax*, *Bcl2*, *Ambra1*, *Map1lc3b*, *Atg7*, and *Becn1* promoters and the *Hprt1* reference), the High-Capacity cDNA-Reverse Transcription Kit, TaqMan Gene Expression Master Mix, and TaqMan probes for specific genes encoding *Hprt1*, *β-actin*, *Gapdh*, *Fas*, *Fasl*, *Bax*, *Bcl2*, *Gsk3b*, *Becn1*, *Atg5*, *Atg7*, *Map1lc3a*, *Map1Lc3b*, *Nup62*, and *Ambra1* were obtained from Thermo Fisher Scientific (Waltham, MA, USA). Quick-gDNA™ MicroPrep and EZ DNA Methylation-Gold™ kits were obtained from ZymoResearch (Irvine, CA, USA).

### Primary Neuronal Cultures of Neocortical Cells

All experiments were conducted on primary cultures of mouse neocortical neurons, which were cultured as previously described [25]. Neuronal cells were isolated from neocortical tissues from Swiss CD1 mouse strain embryos at 15 days of gestation (Charles River, Germany) and plated at a density of 2.0 × 10^5^ cells per cm^2^ on poly-*L*-ornithine-coated multiwell dishes. The cells were suspended in phenol red-free neurobasal medium containing fetal bovine serum, B27, *L*-glutamine, and penicillin–streptomycin antibiotics. The cultures were maintained in a humidified atmosphere (37 °C with 5% (vol/vol) CO_2_), and the experiments were conducted at 7 days *in vitro* (DIV). The number of astrocytes was assessed by the content of intermediate filament glial fibrillary acidic protein (GFAP) and did not exceed 10%, as previously described [26]. All animals used in the research were maintained according to the principles of the Three Rs. The experiments were conducted in compliance with European Union Legislation (Directive 2010/63/EU, amended by Regulation (EU) 2019/1010) and approved by the Bioethics Commission as being compliant with Polish Law (21 August 1997).

### Experimental Models of Hypoxia and Ischemia

#### Hypoxia

The hypoxic conditions were evoked by placing the cultures in a prewarmed and humidified hypoxia modular incubator chamber (Billups-Rothenberg, Inc., CA, USA) with 95% N_2_/5% CO_2_ for 6 h as previously described [24]. The O_2_ level in the chamber was measured with an oxygen analyzer (Greisinger, Germany) and reached less than 0.5%. After 6 h, the neurobasal medium was replaced with standard medium, and the treatment occurred for the next 18 h, i.e., during the reoxygenation period (post-treatment).

#### Ischemia

To induce ischemic conditions, the neurobasal medium was replaced with medium without glucose, and cultures were placed in a prewarmed and humidified hypoxia modular incubator chamber (Billups-Rothenberg, Inc., CA, USA) with 95% N_2_/5% CO_2_ for 6 h. The O_2_ level in the chamber was measured with an oxygen analyzer (Greisinger, Germany) and reached less than 0.5%. After 6 h, the medium without glucose was replaced with standard medium, and the treatment occurred for the next 18 h of the reoxygenation period (post-treatment).

### Treatment

After 6 h of hypoxic/ischemic conditions, i.e., during the reoxygenation period, the culture medium was replaced with standard medium, and treatment with amorfrutin B occurred for the next 18 h (post-treatment). To determine the contribution of apoptotic signaling, we used Z-IETD-FMK (caspase-8 inhibitor; 40 µM), Z-LEHD-FMK (caspase-9 inhibitor; 40 µM), Z-DQMD-FMK (caspase-3/6 inhibitor; 40 µM), TDZD 8 (GSK3β inhibitor; 1 µM), SP600125 (JNK inhibitor; 1 µM) and SB 203580 (p38 MAPK inhibitor; 1 µM), which were administered in the same paradigm as amorfrutin B. To assess the contribution of autophagic signaling, we used temsirolimus (mTOR inhibitor, 1 µM), SBI-0206965 (ULK1 inhibitor, 1 µM), spautin-1 (USP10 and USP13 inhibitor, 1 µM), and MRT68921 dihydrochloride (ULK1 and ULK2 inhibitor, 1 µM), which were also administered in the same paradigm as amorfrutin B. To avoid nonspecific effects, amorfrutin B and all inhibitors were administered at concentrations that did not change the control levels of lactate dehydrogenase (LDH) release under normoxic conditions. According to our previous data [24], we decided to use amorfrutin B at a concentration of 5 µM because it caused the strongest neuroprotective effect under hypoxic and ischemic conditions. The exception was the siRNA experiment in which we used two concentrations of amorfrutin B, i.e., 1 and 5 µM. Moreover, the post-treatment was selected (after 6 h of hypoxia and/or ischemia) to reflect the terms of the potential clinical therapy. Cell cultures were maintained in a humidified incubator (37 °C with 5% (vol/vol) CO_2_), and after 18 h, the biological material was collected for further analysis.

### Assessment of LDH release

Lactate dehydrogenase is an enzyme involved in energy production and is released into the environment in response to cell damage. After the experiments, the cell-free supernatants were collected and used to measure the amount of LDH, as previously described [27]. According to the manufacturer’s instructions, the supernatants were incubated with the appropriate reagent mixture from the Cytotoxicity Detection Kit (Roche, Switzerland) at RT for 30–60 min. The intensity of the red color was measured at a wavelength of 490 nm using an Infinite M200pro microplate reader (Tecan, Männedorf, Switzerland) and was proportional to the amount of cells damaged in response to hypoxia/ischemia. The data were analyzed with i-control software and are presented as a percentage of the control value ± SEM.

### Assessment of the Mitochondrial Membrane Potential

The JC-10 Mitochondrial Membrane Potential Assay Kit (Abcam, Cambridge, Great Britain) was used to measure mitochondrial membrane potential changes in neuronal cells after hypoxia/ischemia and amorfrutin B post-treatment. JC-10 is a lipophilic dye that enters mitochondria and reversibly changes its color from green to orange, which is detected as an increase in membrane potential. JC-10 dye-loading solution was added to the cell plate and incubated with the cells for 1 h at 37 °C as previously described [28]. After adding Assay Buffer B to the dye-loading plate, the fluorescence was monitored at λ_ex_ = 540 nm/λ_em_ = 590 nm and λ_ex_ = 490 nm/λ_em_ = 525 nm with an Infinite M200pro microplate reader (Tecan Mannedorf, Switzerland). The fluorescence intensity was used for the ratio analysis, and the results are presented as a percentage of the control ± SEM.

### Fluorescent Staining with CYTO-ID® and Hoechst 33342

The cultures were grown on coverslips and exposed to hypoxic or ischemic conditions for 6 h at 7 DIV. Neuronal cells were treated with amorfrutin B (tested compound), DMSO (solvent/negative control), or rapamycin (positive control) during the reoxygenation period. After 18 h of treatment, the medium was removed, and the cells were washed with assay buffer. A CYTO-ID green detection kit containing a cationic amphiphilic tracer (CAT) dye and Hoechst 33342 was added to each well, and the plates were incubated at 37 °C in the absence of light. Selective staining with CAT dye allowed the measurement of autophagolysosome accumulation. Fusion between autophagosomes and lysosomes forms autophagolysosomes where degradation of the cytoplasmic contents occurs [29]. Cells with Hoechst 33342 stained bright blue heterochromatin foci, indicating condensed chromatin; an intermediary chromatin remodeling state that relies on formation of apoptosis-associated heterochromatin foci has been suggested to be a marker of apoptosis [6]. After 30 min, the cells were washed with assay buffer, and the coverslips were placed on microscope slides. The stained cells were analyzed by a Leica TCS SP8 WLL confocal laser scanning microscope (DMi8-CS, Leica Microsystem, Wetzlar, Germany) at 60 × magnification as recommended by the manufacturer’s instructions. To quantify the fluorescence signal corresponding to enhanced accumulation of autophagolysosomes, the frequency of the brightest pixels in the region of interest (ROI) was measured. The pixel intensity was assessed using ImageJ software. The mean fluorescence intensity (MFI) was calculated according to the following formula: MFI = MFI of an ROI − MFI of background.

### qPCR Analysis of Apoptosis- and Autophagy-Related Genes

After hypoxia/ischemia and amorfrutin B post-treatment, the total RNA was extracted from neocortical cells using the RNeasy Mini Kit (Qiagen, Hilden, Germany), as previously described [30]. The amount of RNA was assessed at 260 nm and 260/280 nm with a NanoDrop ND-1000 UV–Vis Spectrophotometer (Thermo Fisher Scientific, Waltham, MA, USA). The purity of RNA was accepted when the A260/A280 ratio was ~ 2.0. The RNA extract was used as a cDNA template and reverse-transcribed using a High-Capacity cDNA Reverse Transcription Kit (Thermo Fisher Scientific, USA). The obtained cDNA was amplified using the quantitative polymerase chain reaction (qPCR) method. Both reverse transcription and qPCR were run on a CFX 96 Real-Time PCR Detection System (Bio–Rad, Hercules, CA, USA). For amplification, we used a solution containing 1 µl cDNA, 10 μl FastStart Universal Probe Master, 1 μl TaqMan Gene Expression Assay mix, and 8 μl RNase-free water. The chosen TaqMan Gene Expression Assays (Thermo Fisher Scientific, USA) were specific for apoptosis-related (*Fas*, *Fasl*, *Bax*, *Bcl2*, and *Gsk3b*) and autophagy-related genes (*Becn1*, *Atg5*, *Atg7*, *Map1lc3a*, *Map1lc3b*, *Nup62*, and *Ambra1*). The qPCR procedure consisted of a series of following temperature changes: 2 min at 50 °C and 10 min at 95 °C, followed by 40 cycles of 15 s at 95 °C and 1 min at 60 °C. The qPCR process was performed using a CFX96 Real-Time system (Bio–Rad, USA), and the data were analyzed using the delta Ct method. The following algorithms, i.e., geNorm, NormFinder, BestKeeper and delta Ct, selected *Hprt1* (instead of *β-actin* and *Gapdh*) as the most stable reference gene. The results are presented as fold changes ± SEM.

### Enzyme-Linked Immunosorbent Assays for Apoptosis- and Autophagy-Related Proteins

The neocortical cells that had undergone hypoxia/ischemia and amorfrutin B post-treatment were lysed and sonicated in ice-cold RIPA lysis buffer with a protease inhibitor cocktail. After centrifugation (15,000×g for 20 min at 4°C), the supernatants were collected, and the protein concentration was assessed using the Bradford method. The expression levels of apoptosis- (FAS, FASL, BAX, BCL2, and GSK3β) and autophagy-related (BECN1, ATG7, MAP1LC3B, and AMBRA1) proteins were assessed using enzyme-linked immunosorbent mouse-specific assays (Bioassay Technology Laboratory, China; ELK Biotechnology, China). The standards and samples were attached to the biotin-conjugated polyclonal antibodies in each well. Then, streptavidin-HRP was added, and all wells were washed with buffer. The reaction was completed by adding the substrate and acidic stop solution, which resulted in a color change from yellow to blue. The absorbance was measured at 450 nm using an Infinite M200PRO microplate reader, and the values were correlated with the amounts of the specific proteins. The data are presented as a percentage of the control value ± SEM and pg/mg of total protein.

### Silencing of Autophagy-Related Genes Using Specific siRNAs

To inhibit the expression of the *Becn1* and *Atg7* genes in neocortical cells, specific *Becn1* and *Atg7* siRNAs (Santa Cruz Biotechnology Inc., USA) were used as previously described [31]. The siRNAs (at a final concentration of 50 nM) containing transfection reagent (INTERFERin) were applied in antibiotic-free medium for 7 h. After transfection, the culture medium was replaced with standard medium containing antibiotics, and neuronal cells were incubated until the next day of the experiment. Control cells were transfected with negative siRNA, i.e., scrambled sequence that did not lead to specific degradation of any known cellular mRNA. To verify the mRNA silencing, we measured the expression levels of specific mRNAs. As previously described, *Becn1* and *Atg7* expression levels decreased by 68% and 57%, respectively [31].

### Assessment of the Methylation of Apoptosis- and Autophagy-Related Gene Promoters

To extract genomic DNA from neuronal cells, a QuickgDNA™ MicroPrep kit (Zymo Research, Irvine, CA) was used according to the manufacturer’s protocol. The quantity of DNA was rated spectrophotometrically at 260 nm and 260/280 nm using a NanoDrop ND-1000 UV–Vis Spectrophotometer (Thermo Fisher Scientific, USA). Next, the EpiTect Bisulfite kit (Qiagen, Hilden, Germany) was applied to denature DNA and perform bisulfite conversion. To detect changes in DNA methylation status, the EpiTect MethyLight PCR kit (Qiagen, Hilden, Germany) and sets of TaqMan probes were used. Sets were designed for the bisulfite-converted DNA sequences and represented: (i) fully methylated and fully unmethylated probes for the *Bax*, *Bcl2*, *Ambra1*, *Map1lc3b*, *Atg7*, and *Becn1* promoters, (ii) the internal reference set for the *Hprt1* gene to control for input DNA. Quantitative real-time polymerase chain reaction (MethyLight) was performed as previously described [30], and the EpiTect MethyLight assays enabled the calculation of the degree of methylation according to the following formula: ΔΔCt = methylated signal (Ct target gene − Ct *Hprt1*) − unmethylated signal (Ct target gene − Ct *Hprt1*). The results are presented as the methylation rate ± SEM.

### qPCR Analysis of Specific Apoptosis-Focused miRNAs Determined Using the RT2 Profiler PCR Array

miRNA was isolated from neuronal cell cultures using the miRNeasy Mini Kit (Qiagen, Hilden, Germany) and quantified spectrophotometrically at 260 nm and 260/280 nm with a NanoDrop ND-1000 UV–Vis Spectrophotometer (Thermo Fisher Scientific, USA). Reverse transcription to cDNA was performed using a miRCURY LNA RT Kit (Qiagen, Hilden, Germany). A reaction mixture consisting of our template miRNA and reverse transcription master mix was incubated for 60 min at 42 °C and 5 min at 95 °C. To analyze apoptosis-focused miRNA expression, the MiRCURY LNA miRNA Focus Panel (Qiagen, Hilden, Germany) was used according to the manufacturer’s protocol. Both RT and qPCR were performed with the CFX96 Real-Time System (Bio–Rad, Hercules, CA, USA). *Ct* values were analyzed using GeneGlobe Qiagen web-based software, and the reference genes were U6 snRNA (v2), 5S rRNA, RNU5G, and RNU1A1. Fold changes are presented as heatmaps, which were visualized in Python 3 using the Seaborn module.

### Statistical Analysis

The statistical analysis of the data was performed using raw data measured as the absorbance or fluorescence units per well containing 50,000 cells for the LDH and JC-10 assay; the fluorescence units per 1.5 million cells for qPCR; and the pg of FAS, FASL, BAX, BCL2, GSK3β, ATG7, BECN1, MAP1LC3B, and AMBRA1 per mg of total protein for the ELISAs. To determine overall significance, an analysis of variance (ANOVA) and Levene’s test of homogeneity of variances were used. The differences between the control and experimental groups were defined with a post hoc Newman–Keuls test. Significant differences were marked in the following ways: ^*^p < 0.05, ^**^p < 0.01, and ^***^p < 0.001 (compared to the control groups), ^#^*p* < 0.05, ^##^*p* < 0.01, and ^###^*p* < 0.001 (compared to the cultures exposed to hypoxia), ^^^*p* < 0.05, ^^^^*p* < 0.01, and ^^^^^*p* < 0.001 (compared to the cultures exposed to ischemia), and ^$$$^*p* < 0.001 (compared to negative control, scrambled siRNA). The results for qPCR and ELISAs are expressed as the mean ± SEM. The results for LDH release and JC-10 and fluorescence intensity are presented as the percentage of the control ± SEM. In case of LDH release measurement and JC-10 test, the results were obtained from 3 independent experiments, and the number of replicates ranged from 8 to 12. The results from qPCR, ELISA, and gene specific methylation level were obtained from 3 independent experiments, and the number of replicates ranged from 5 to 6. The fluorescence intensity was measured based on 5 replicates. In case of microarray analysis, the results are based on 5 replicates for each experimental group. The effect of amorfrutin B administration on cells under normoxic conditions is presented as the supplementary material (Table S1 and Fig. S1).

## Results

### Apoptosis- and Autophagy-Related Pathways Are Involved in Hypoxia- and Ischemia-Induced Neuronal Cell Death

**Fig. 1:**
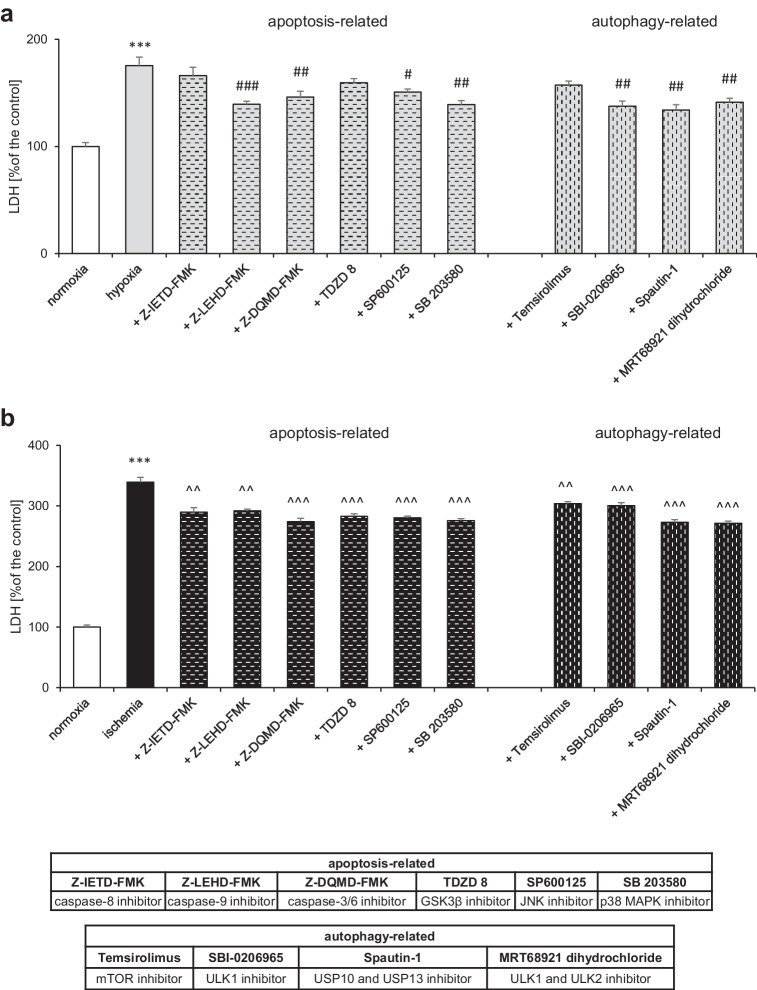
Hypoxia and ischemia evoked cell death *via* apoptosis- and autophagy-related pathways in primary neocortical cell cultures. Post-treatment with apoptosis- and autophagy-related inhibitors of caspases, kinases, and hydrolases decreased hypoxia- (**a**) and ischemia-induced (**b**) LDH release depending on the model. The results are presented as a percentage of the control ± SEM of 3 independent experiments, consisting of 8–12 replicates per group. ^***^p < 0.001 compared to the control group, ^#^*p* < 0.05, ^##^*p* < 0.01, ^###^*p* < 0.001 compared to the cultures exposed to hypoxia, ^^^^*p* < 0.01, ^^^^^*p* < 0.001 compared to the cultures exposed to ischemia

In the present study, 6 h of hypoxia followed by 18 h of reoxygenation induced LDH release up to 176% of the normoxic value. Post-treatment with apoptosis-related inhibitors of caspases-9 and -3/6 (Z-LEHD-FMK and Z-DQMD-FMK, respectively) as well as JNK and p38 MAPK kinases (SP600125, SB 203,580) resulted in LDH activity decreases to 139%, 146%, 151%, and 139% of the control, respectively. In turn, the administration of Z-IETD-FMK (caspase-8 inhibitor) and TDZD 8 (GSK3β inhibitor) did not have a statistically significant effect on LDH levels under hypoxic conditions. Except for temsirolimus (mTOR inhibitor), specific inhibitors of kinases ULK1 and ULK1/ULK2 (SBI-0206965 and MRT68921 dihydrochloride) as well as hydrolases USP10/USP13 (Spautin-1) involved in the process of autophagy decreased LDH release to 138%, 141%, and 134% of the control, respectively (Fig. 1a).

A model of 6-h ischemia followed by 18 h of reoxygenation resulted in an increase in the activity of LDH to 339% of the normoxic value. All apoptosis-related inhibitors of caspase-8, -9, -3/6, GSK3β, JNK, and p38 MAPK (Z-IETD-FMK, Z-LEHD-FMK, Z-DQMD-FMK, TDZD 8, SP600125, and SB 203580) reduced LDH activity to 290%, 292%, 274%, 283%, 280%, and 276% of the control, respectively. In addition, specific inhibitors of mTOR, ULK1, USP10/USP13, and ULK1/ULK2 involved in the process of autophagy, i.e., temsirolimus, SBI-0206965, spautin-1, and MRT68921 dihydrochloride, decreased LDH release to 304%, 301%, 273%, and 271% of the control, respectively (Fig. 1b).

### Post-treatment with Amorfrutin B Partially Reversed Hypoxia- and Ischemia-Reduced Mitochondrial Membrane Potential

**Fig. 2:**
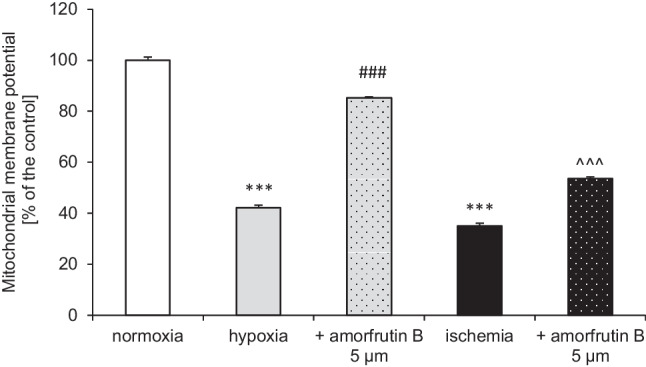
Post-treatment with amorfrutin B partially reversed hypoxia- and ischemia-induced loss of mitochondrial membrane potential. The results are presented as a percentage of the control ± SEM of 3 independent experiments, consisting of 8–12 replicates per group. ^***^p < 0.001 compared to the control group, ^###^*p* < 0.001 compared to the cultures exposed to hypoxia, ^^^^^*p* < 0.001 compared to the cultures exposed to ischemia

In this study, 6 h of hypoxia/ischemia and 18 h of reoxygenation led to mitochondrial membrane potential decreases to 42% and 35% of the normoxic value, respectively. Post-treatment with amorfrutin B partially reversed the effects of hypoxia and ischemia, i.e., the mitochondrial membrane potential reached 85% and 54% of the control (Fig. 2).

### Post-treatment with Amorfrutin B Reduced the Formation of Apoptosis-Associated Heterochromatin Foci and Autophagolysosomes in Hypoxia/Ischemia-Exposed Neocortical Cells

**Fig. 3:**
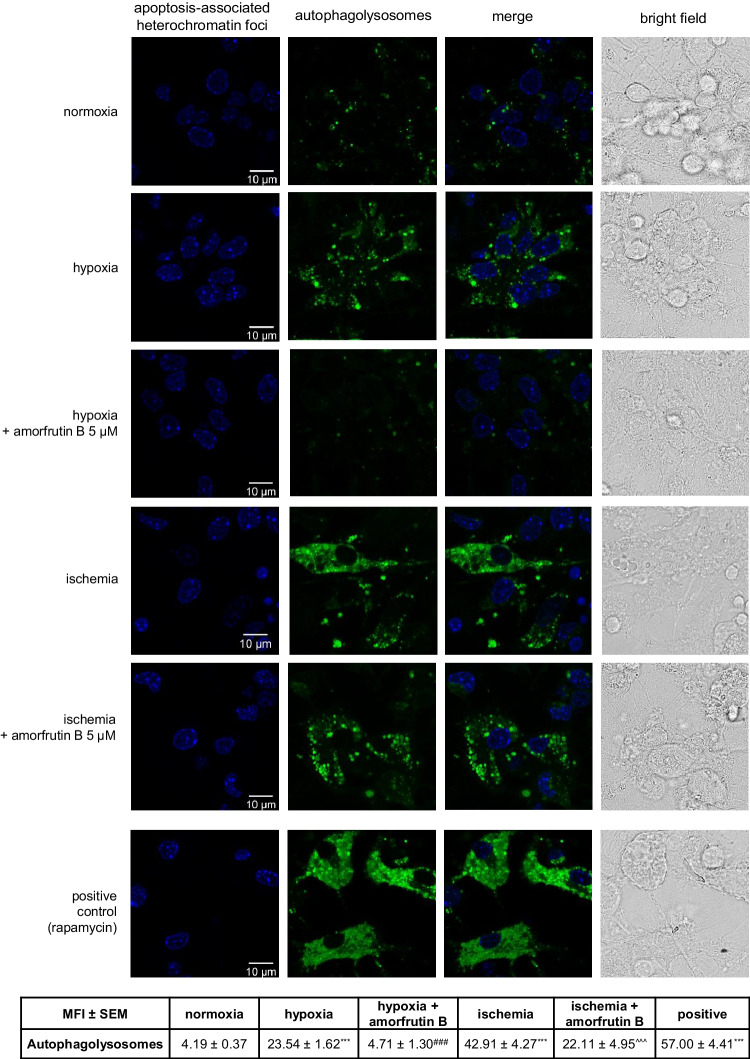
Amorfrutin B post-treatment reduced the formation of apoptosis-associated heterochromatin foci (bright blue) and autophagolysosomes (green) in neuronal cells undergoing hypoxia and ischemia. Heterochromatin foci and autophagolysosomes were visualized with the use of Hoechst 33342 and CAT dye, respectively. The bright-field images were included. Positive control indicated the formation of autophagolysosomes after treatment with rapamycin. The table shows results presented as MFI (mean fluorescence intensity) ± SEM. ^***^p < 0.001 compared to the control group, ^###^*p* < 0.001 compared to the cultures exposed to hypoxia, ^^^^^*p* < 0.001 compared to the cultures exposed to ischemia. The number of replicates in each group was 5

Both hypoxia and ischemia induced apoptosis in neuronal cell cultures, which was manifested by the formation of apoptosis-associated heterochromatin foci visible as bright blue condensed chromatin labeled with Hoechst 33342. Post-treatment with amorfrutin B reduced chromatin remodeling. The CYTO-ID labeling and intensity quantification method showed the formation of autophagolysosomes in neurons undergoing hypoxia and ischemia, which was indicated by increased levels of MFI from 4.19 in normoxia to 23.54 in hypoxia and 42.91 in ischemia. Administration of amorfrutin B resulted in inhibition of autophagolysosome formation in the case of hypoxia (MFI reached 4.71); however, the compound only partially reversed autophagolysosome formation in the case of ischemia (MFI reached 22.11). Our study included a positive control indicating the formation of autophagolysosomes after treatment with the autophagy inducer—rapamycin (Fig. 3).

### Post-treatment with Amorfrutin B Normalized the Expression Levels of Apoptosis-Related Genes and Proteins in Neuronal Cells Exposed to Hypoxia/Ischemia

**Fig. 4:**
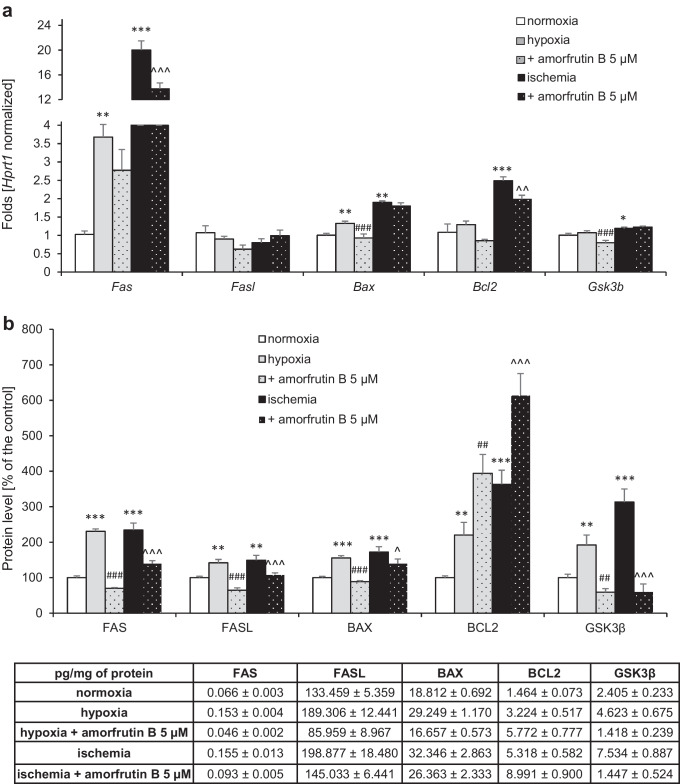
Post-treatment with amorfrutin B normalized the expression levels of apoptosis-related genes (**a**) and proteins (**b**) in neuronal cells exposed to hypoxia or ischemia. The results are presented as a fold change in case of qPCR and percentage of the control or pg/mg of protein ± SEM in case of ELISA. There were 3 independent experiments, consisting of 5–6 replicates per group. ^*^p < 0.05, ^**^p < 0.01, and ^***^p < 0.001 compared to the control group, ^##^*p* < 0.01, ^###^*p* < 0.001 compared to the cultures exposed to hypoxia, ^^^*p* < 0.05, ^^^^*p* < 0.01, ^^^^^*p* < 0.001 compared to the cultures exposed to ischemia

In neuronal cell cultures exposed to hypoxia, expression levels of apoptosis-related genes, i.e., *Fas* (2.68-fold increase) and *Bax* (0.33-fold increase) were increased, compared to the control group. Amorfrutin B administered 6 h after the initiation of hypoxic damage diminished the expression levels of *Bax* and *Gsk3β* to 0.93- and 0.80-fold of the control values, respectively. Ischemia induced increases in the expression levels of the following genes: *Fas* (19.02-fold increase), *Bax* (0.90-fold increase), *Bcl2* (1.49-fold increase), and *Gsk3β* (0.19-fold increase) compared to normoxia. In this model, post-treatment with amorfrutin B decreased the expression levels of *Fas* and *Bcl2* to 13.74- and 1.98-fold of the control values, respectively. The exception was *Fasl*, whose mRNA expression level did not change after hypoxia, ischemia, or amorfrutin B post-treatment (Fig. 4a).

ELISA analyses revealed that the levels of all apoptosis-related proteins were enhanced due to both hypoxic and ischemic conditions. Under hypoxia, FAS, FASL, BAX, BCL2, and GSK3β protein levels increased to 231%, 142%, 156%, 220%, and 192% (equal to 0.15, 189.36, 29.25, 3.22, 4.62 pg per mg of total protein) of the normoxic value, respectively. In response to amorfrutin B post-treatment after hypoxia, the protein expression levels decreased to 70% (0.05 pg/mg) in case of FAS, 64% (85.96 pg/mg) in case of FASL, 89% (16.66 pg/mg) in case of BAX, and 59% (1.42 pg/mg) in case of GSK3β compared to the normoxic value. In turn, the BCL2 protein level increased to 394% (5.77 pg/mg) of the control after administration of amorfrutin B. Similarly, the protein expression levels of FAS, FASL, BAX, BCL2, and GSK3β increased to 234%, 149%, 172%, 363%, and 313% (equal to 0.16, 198.88, 32.35, 5.32, 7.53 pg per mg of total protein) of the control in response to ischemic conditions. Amorfrutin B post-treatment after ischemia reduced the protein levels of FAS (140%, 0.09 pg/mg), FASL (109%, 145.03 pg/mg), BAX (140%, 26.36 pg/mg), and GSK3β (60%, 1.45 pg/mg). However, the expression level of BCL2 increased to 614% (8.99 pg/mg) of the control after exposure to the compound (Fig. 4b).

### Amorfrutin B Interfered with the Autophagy-Related mRNA and Protein Expression Levels After Exposure to Hypoxia and Ischemia

**Fig. 5:**
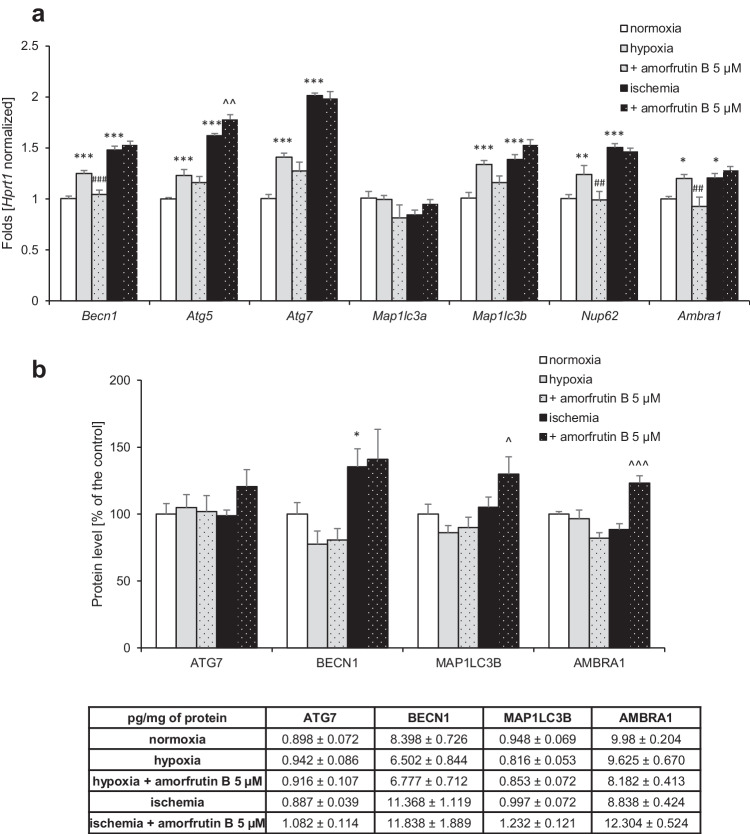
Amorfrutin B post-treatment affected the expression levels of autophagy-related genes (**a**) and proteins (**b**), as measured using qPCR and ELISA. The results are presented as a fold change in case of qPCR and percentage of the control or pg/mg of protein ± SEM in case of ELISA. There were 3 independent experiments, consisting of 5–6 replicates per group. ^*^p < 0.05, ^**^p < 0.01, and ^***^p < 0.001 compared to the control group, ^##^*p* < 0.01, ^###^*p* < 0.001 compared to the cultures exposed to hypoxia, ^^^*p* < 0.05, ^^^^*p* < 0.01, ^^^^^*p* < 0.001 compared to the cultures exposed to ischemia

In our research, qPCR analysis showed that all autophagy-related genes were excessively expressed due to hypoxia and ischemia, except *Map1lc3a*, whose expression remained unchanged in both models. Under hypoxic conditions, there were increases in the expression levels of *Becn1* (0.25-fold increase), *Atg5* (0.23-fold increase), *Atg7* (0.4-fold increase), *Map1lc3b* (0.34-fold increase), *Nup62* (0.24-fold increase), and *Ambra1* (0.2-fold increase). Exposure to amorfrutin B 6 h after hypoxic injury resulted in a decrease in the mRNA expression levels of *Becn1* to 1.05-fold, *Nup62* to 0.99-fold, and *Ambra1* to 0.93-fold. In response to ischemia, mRNA expression of *Becn1* (0.48-fold increase), *Atg5* (0.62-fold increase), *Atg7* (1.02-fold increase), *Map1lc3b* (0.39-fold increase), *Nup62* (0.5-fold increase), and *Ambra1* (0.21-fold increase) was upregulated. Amorfrutin B post-treatment only increased the expression of *Atg5* to 1.78-fold, and the expression levels of other genes did not change (Fig. 5a).

ELISA analyses showed that only the BECN1 expression level increased to 135% (equal to 11.37 pg per mg of total protein) of the control under ischemic conditions and remained unchanged under hypoxic conditions. The levels of other autophagy-related proteins (ATG7, MAP1LC3B, and AMBRA1) did not differ significantly between the hypoxia/ischemia conditions and the control normoxia. Amorfrutin B did not affect the levels of any autophagy-related proteins under hypoxic conditions. However, post-treament with amorfrutin B after 6 h of ischemia increased the protein levels of MAP1LC3B (130%, 1.23 pg/mg) and AMBRA1 (123%, 12.30 pg/mg), while the expression levels of other proteins (BECN1 and ATG7) remained unchanged (Fig. 5b). During ischemia, overexpression of MAP1LC3B and AMBRA1 after amorfrutin B post-treatment could be related to other than autophagy-related functions of these proteins. An increase in MAP1LC3B expression level can be associated with neuronal structure improvement [32], whereas overexpression of AMBRA1 may predispose cells to avoid apoptosis and promote their survival [33].

### Silencing of Becn1 and/or Atg7 Reduced the Neuroprotective effects of Amorfrutin B Against Hypoxia- and Ischemia-Induced Cell Damage

**Fig. 6:**
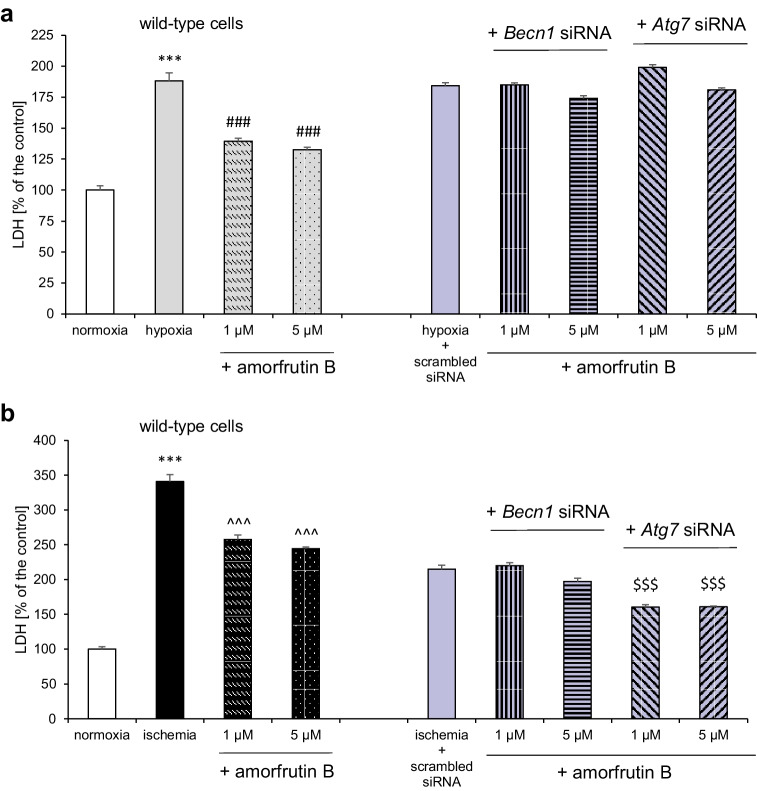
Silencing of *Becn1* and/or *Atg7* reduced the neuroprotective effects of amorfrutin B against hypoxia- and ischemia-induced cell damage. In *Atg7* siRNA-transfected cells, amorfrutin B lost its neuroprotective capacity under hypoxic (**a**) but not ischemic (**b**) conditions. The results were normalized to the absorbance in negative control (scrambled siRNA). The results are presented as a percentage of the control ± SEM of 3 independent experiments, consisting of 8–12 replicates per group. ^***^p < 0.001 compared to normoxia, ^###^*p* < 0.001 compared to the cultures exposed to hypoxia, ^^^^^*p* < 0.001 compared to the cultures exposed to ischemia, ^$$$^*p* < 0.001 compared to negative control

In wild-type cells subjected to hypoxia and ischemia, amorfrutin B (1 and 5 µM) protected neuronal cell cultures from cell death, as we previously described [24]. In *Becn1* and *Atg7* siRNA-transfected cells, amorfrutin B lost its neuroprotective potential and did not prevent hypoxia-induced LDH release in comparison to the negative control (Fig. 6a). In the case of ischemia, the silencing of *Becn1* expression with specific siRNA also resulted in the loss of the neuroprotective properties of amorfrutin B. However, in *Atg7* siRNA-transfected cells, amorfrutin B (1 and 5 µM) still protected cells from ischemia-induced cell death, as evidenced by the decrease in the LDH parameter to 161% compared to the negative control (Fig. 6b).

The negative control siRNA (scrambled siRNA) that is a control for specific/positive siRNAs may impair the proliferation and viability of cell cultures [34]. Therefore, exposure to ischemia of scrambled siRNA-treated cells would result in smaller effect (low cell density) than exposure to ischemia of wild-type cells (high cell density).

### Changes in the Methylation Rates of Apoptosis- and Autophagy-Related Genes in Response to Hypoxia/Ischemia Conditions and Amorfrutin B Post-treatment

**Fig. 7:**
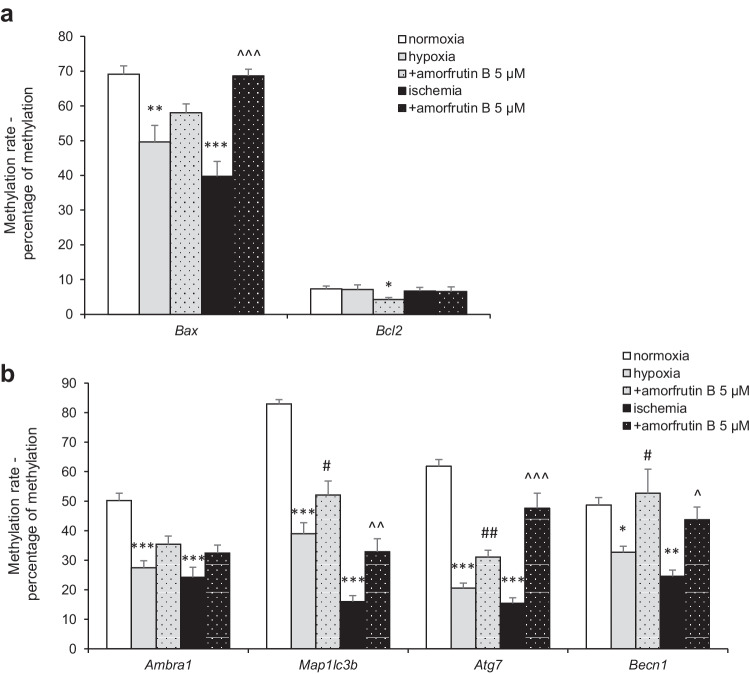
Alterations in apoptosis- (**a**) and autophagy-related (**b**) gene-specific methylation after hypoxia or ischemia and amorfrutin B post-treatment. The results are presented as the mean ± SEM. There were 3 independent experiments consisting of 5 replicates per group. ^*^p < 0.05, ^**^p < 0.01, and ^***^p < 0.001 compared to the control group, ^#^*p* < 0.05, ^##^*p* < 0.01 compared to the cultures exposed to hypoxia, ^^^*p* < 0.05, ^^^^*p* < 0.01, ^^^^^*p* < 0.001 compared to the cultures exposed to ischemia

The methylation rates of apoptosis-related genes were analyzed under normoxic conditions and reached 69% for *Bax* and 7% for *Bcl2*. In response to hypoxia and ischemia, the *Bax* methylation decreased to 50% and 40%, respectively. The methylation rate of *Bcl2* did not change in response to hypoxia and ischemia. Post-treatment with amorfrutin B after 6 h of hypoxia did not change the *Bax* methylation rate; however, it increased to 69% in neuronal cells subjected to 6 h of ischemia. Moreover, amorfrutin B administration decreased the methylation rate of *Bcl2* observed during hypoxia to 4% (Fig. 7a).

In the case of autophagy-related genes, under normoxic conditions, the methylation rates of *Ambra1*, *Map1lc3b*, *Atg7,* and *Becn1* reached values of 50%, 83%, 62%, and 49%, respectively. In response to 6-h hypoxia and 6-h ischemia, the methylation rates of all genes were reduced: the *Ambra1* methylation rate diminished to 27% (hypoxia) and 24% (ischemia), the *Map1lc3b* methylation rate decreased to 39% (hypoxia) and 16% (ischemia), the *Atg7* methylation rate decreased to 21% (hypoxia) and 15% (ischemia), and the *Becn1* methylation rate was reduced to 33% (hypoxia) and 25% (ischemia). Post-treatment with amorfrutin B caused increases in the methylation of all studied genes, except for *Ambra1*. Following exposure to amorfrutin B, the methylation rate of *Map1lc3b* increased to 52% (hypoxia) and 33% (ischemia), and the methylation rate of *Atg7* increased to 31% (hypoxia) and 48% (ischemia). Moreover, the methylation rate of *Becn1* increased to 53% in the case of hypoxia and 44% in the case of ischemia (Fig. 7b).

### Effects of Amorfrutin B Post-treatment on the Expression of Apoptosis-Focused miRNAs Under Hypoxic and Ischemic Conditions as Determined Using an RT2 Profiler PCR Array

The RT^2^ Profiler PCR Array enabled the analysis of the expression profiles of 84 apoptosis-focused miRNAs in neuronal cell cultures subjected to hypoxia or ischemia and amorfrutin B post-treatment.

#### Hypoxia Dysregulated the Expression of 34 Apoptosis-Focused miRNAs (Fig. 8a)

**Fig. 8:**
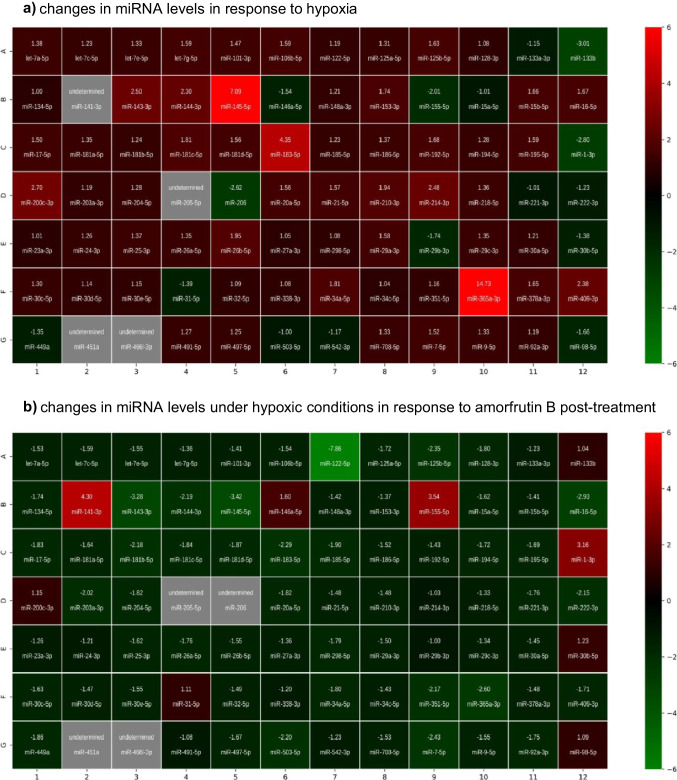

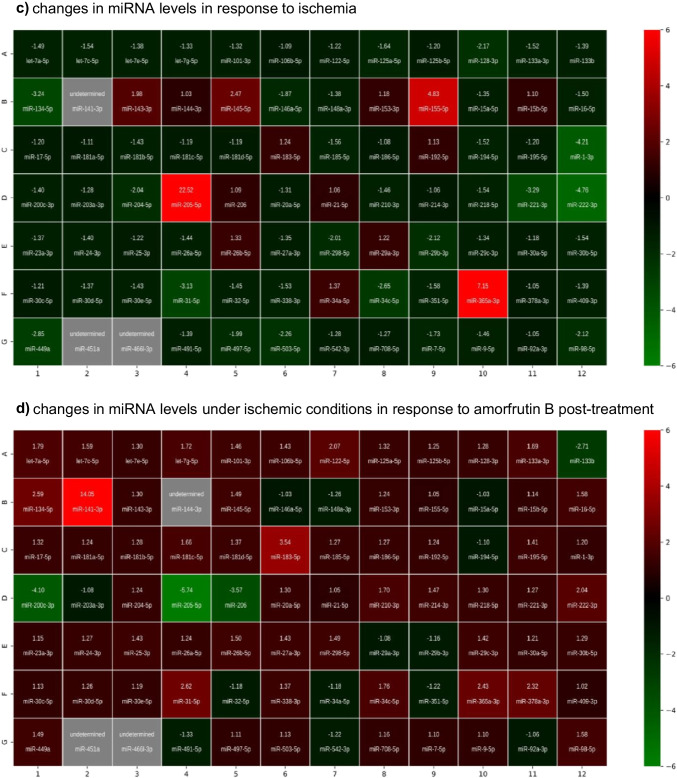
Amorfrutin B post-treatment dysregulated the expression levels of apoptosis-focused miRNAs in neuronal cell cultures undergoing hypoxia and ischemia. The results were determined by microarray analyses and are shown as heatmaps of 84 miRNAs. The results are based on 5 replicates for each experimental group. The cell’s color indicates the fold change of miRNA in the corresponding cell range. Downregulated miRNAs are shown in green, upregulated miRNAs are shown in red, and undetermined miRNAs are shown in gray

(i) Seven miRNAs were downregulated (shown in green): *miR-133b*, *miR-146a-5p*, *miR-155-5p*, *miR-1-3p*, *miR-206*, *miR-29b-3p*, and *miR-98-5p*.
(ii) Twenty-seven miRNAs were upregulated (shown in red): *let-7 g-5p*, *miR-106b-5p*, *miR-125b-5p*, *miR-143-3p*, *miR-144-3p*, *miR-145-5p*, *miR-153-3p*, *miR-15b-5p*, *miR-16-5p*, *miR-17-5p*, *miR-181c-5p*, *miR-181d-5p*, *miR-183-5p*, *miR-192-5p*, *miR-195-5p*, *miR-200c-3p*, *miR-20a-5p*, *miR-21-5p*, *miR-210-3p*, *miR-214-3p*, *miR-26b-5p*, *miR-29a-3p*, *miR-34a-5p*, *miR-365a-3p*, *miR-378a-3p*, *miR-409-3p*, and *miR-7-5p*.

#### Under Hypoxic Conditions, Amorfrutin B Post-treatment Dysregulated 51 Apoptosis-Focused miRNAs (Fig. 8b)

(i) Forty-seven miRNAs were downregulated (shown in green): *let-7a-5p*, *let-7c-5p*, *let-7e-5p*, *miR-106b-5p*, *miR-122-5p*, *miR-125a-5p*, *miR-125b-5p*, *miR-128-3p*, *miR-134-5p*, *miR-143-3p*, *miR-144-3p*, *miR-145-5p*, *miR-15a-5p*, *miR-16-5p*, *miR-17-5p*, *miR-181a-5p*, *miR-181b-5p*, *miR-181c-5p*, *miR-181d-5p*, *miR-183-5p*, *miR-185-5p*, *miR-186-5p*, *miR-194-5p*, *miR-195-5p*, *miR-203a-3p*, *miR-204-5p*, *miR-20a-5p*, *miR-221-3p*, *miR-222-3p*, *miR-25-3p*, *miR-26a-5p*, *miR-26b-5p*, *miR-298-5p*, *miR-29a-3p*, *miR-30c-5p*, *miR-30e-5p*, *miR-34a-5p*, *miR-351-5p, miR-365a-3p*, *miR-409-3p*, *miR-449a*, *miR-497-5p*, *miR-503-5p*, *miR-708-5p*, *miR-7-5p*, *miR-9-5p*, and *miR-92a-3p*.
(ii) Four miRNAs were upregulated (shown in red): *miR-141-3p*, *miR-146a-5p*, *miR-155-5p*, and *miR-1-3p*.

A number of miRNAs were oppositely regulated by hypoxia and amorfrutin B. Many hypoxia-upregulated miRNAs were downregulated in response to amorfutin B, namely *miR-106b-5p*, *miR-125b-5p*, *miR-143-3p*, *miR-144-3p*, *miR-145-5p*, *miR-16-5p*, *miR-17-5p*, *miR-181c-5p*, *miR-181d-5p*, *miR-183-5p*, *miR-195-5p*, *miR-20a-5p*, *miR-26b-5p*, *miR-29a-3p*, *miR-34a-5p*, *miR-365a-3p*, *miR-409-3p*, and *miR-7-5p*. Moreover, amorfrutin B enhanced the expression of several miRNAs that were diminished during the course of hypoxia, i.e., *miR-146a-5p*, *miR-155-5p*, and *miR-1-3p*.

#### Ischemia Dysregulated the Expression of 31 Apoptosis-Focused miRNAs (Fig. 8c)

(i) Twenty-six miRNAs were downregulated (shown in green): *let-7c-5p*, *miR-125a-5p*, *miR-128-3p*, *miR-133a-3p*, *miR-134-5p*, *miR-146a-5p*, *miR-16-5p*, *miR-185-5p*, *miR-194-5p*, *miR-1-3p*, *miR-204-5p*, *miR-218-5p*, *miR-221-3p*, *miR-222-3p*, *miR-298-5p*, *miR-29b-3p*, *miR-30b-5p*, *miR-31-5p*, *miR-338-3p*, *miR-34c-5p*, *miR-351-5p*, *miR-449a*, *miR-497-5p*, *miR-503-5p*, *miR-7-5p*, and *miR-98-5p*.
(ii) Five miRNAs were upregulated (shown in red): *miR-143-3p*, *miR-145-5p*, *miR-155-5p*, *miR-205-5p*, and *miR-365a-3p*.

#### Under Ischemic Conditions, Amorfrutin B Post-treatment Dysregulated 22 Apoptosis-Focused miRNAs (Fig. 8d)

(i) Four miRNAs were downregulated (shown in green): *miR-133b*, *miR-200c-3p*, *miR-205-5p*, and *miR-206*.
(ii) Eighteen miRNAs were upregulated (shown in red): *let-7a-5p*, *let-7c-5p*, *let-7g-5p*, *miR-122-5p*, *miR-133a-3p*, *miR-134-5p*, *miR-141-3p*, *miR-16-5p*, *miR-181c-5p*, *miR-183-5p*, *miR-210-3p*, *miR-222-3p*, *miR-26b-5p*, *miR-31-5p*, *miR-34c-5p*, *miR-365a-3p*, *miR-378a-3p*, and *miR-98-5p*.

Ischemic conditions and amorfrutin B oppositely regulated certain miRNAs. These include amorfutin B-downregulated miRNA, which was upregulated during ischemia, i.e., *miR-205-5p*. Amorfrutin B also increased the expression levels of some miRNAs that had reduced expression during ischemia, such as *let-7c-5p*, *miR-133a-3p*, *miR-134-5p*, *miR-16-5p*, *miR-222-3p*, *miR-31-5p*, *miR-34c-5p*, and *miR-98-5p*.

## Discussion

Amorfrutin B is a selective modulator of the PPARγ receptor, which we recently identified as an effective neuroprotective compound that protects brain neurons from hypoxic and ischemic damage when applied as a 6-h delayed post-treatment [24]. Previously, we recognized amorfrutin B as a selective PPARγ modulator that activated PPARγ in the neuronal cells undergoing hypoxia or ischemia and in this way evoked neuroprotection; amorfrutin B promoted mitochondrial integrity and was capable to inhibit ROS activity and ROS-mediated DNA damage. Since the mechanisms of amorfrutin B-attributed neuroprotection are only partially recognized, this study aimed to assess the apoptosis- and autophagy-related effects of the compound in cellular models of brain hypoxia and ischemia. The adequacy of the implemented models has been confirmed by the use of specific inhibitors targeting apoptosis (caspase-8, -9, -3/6, GSK3β, JNK, p38 MAPK) and autophagy (mTOR, ULK1, ULK2, USP10, USP13) that diminished hypoxia/ischemia-induced neurotoxicity. Moreover, the contribution of apoptosis- and autophagy-dependent signaling to the development of hypoxic/ischemic injuries in brain neurons has been supported by other studies, including ours [28, 35–49].

Although the neuroprotective potential of PPARγ agonists for the treatment of brain hypoxia and stroke is well-accepted, only certain agonists, namely thiazolidinediones, appeared effective when applied as a post-treatment [50–52]. However, thiazolidinediones such as troglitazone were found to cause hepatotoxicity, and rosiglitazone and pioglitazone increased the risk of heart failure [53]. The unique property of amorfrutin B to act as a selective PPARγ modulator is that it covers a partial agonism and/or a partial antagonism of the receptor that favors a safer pharmacological profile than full PPARγ agonism [54]. There is no study, except for ours, showing the neuroprotective effect of post-treatment with a selective PPARγ modulator against hypoxia or ischemia. Telmisartan, which is a selective PPARγ modulator that binds to the receptor in a different way than thiazolidinediones, suppressed cerebral injury in a murine model of transient focal ischemia, but it was used as a pre-treatment [55]. Since amorfrutin B appeared to exert neuroprotection when applied with a 6-h delay from initiating hypoxia or ischemia, one may assume that amorfrutin B has the ability to adjust to clinical standards without evoking severe side effects.

We demonstrated for the first time that post-treatment with amorfrutin B prevented neuronal apoptosis in terms of the loss of mitochondrial membrane potential, heterochromatin foci formation, and the expression of specific genes and proteins, such as *Fas*/FAS, FASL, *Bax*/BAX, *Bcl2*/BCL2, and *Gsk3b*/GSK3β. The expression levels of all studied apoptosis-related genes and proteins were decreased in response to amorfrutin B, both during hypoxia and ischemia, except for the expression of anti-apoptotic BCL2, which was increased. Human studies showed the upregulation of the BCL2 expression in the brains of AD (Alzheimer’s disease) patients [56–58]. Also, BCL2 expression was upregulated in the brains of HD (Huntington’s disease) patients with longer disease duration [59]. In both cases, the increase in BCL2 expression is likely the result of the activation of a defense mechanism and attempt to survive. In our study, after post-treatment with amorfrutin B, the methylation rate of the pro-apoptotic *Bax* gene was inversely correlated with the protein level, which explained the decrease in the BAX/BCL2 ratio as a result of *Bax* hypermethylation. Previously, we showed altered methylation levels of *Bcl2* and *Bax* genes that were correlated with an increased BAX/BCL2 ratio in response to neurotoxic action of triclocarban [26]. Another study from our group showed that benzophenone-3-induced neurotoxicity involved *Bax* hypomethylation and *Bcl2* hypermethylation, which confirmed a key role of DNA methylation in the regulation of the expression levels of apoptosis-related factors [30].

The study showed that the mechanisms of protective action of amorfrutin B also involved an inhibition of autophagy, as evidenced by diminished autophagolysosome formation and loss of neuroprotective properties of amorfrutin B after the silencing of *Becn1* and *Atg7* in cells undergoing hypoxia or after the silencing of *Becn1* in cells undergoing ischemia. In many stroke models determining final fate of neurons, accumulation of autophagosomes and activation of lysosomal function is detected [60]. Autophagolysosomes arise from autophagosome and lysosome fuse allowing degradation of the cytoplasmic contents [29], and by inhibiting autophagolysosomes formation, amorfrutin B would have elicited neuroprotection. The results are in line with the observation that a selective neuronal deletion of *Atg7* in the neonatal brain inhibited hypoxia–ischemia-induced autophagy and caused neuroprotection [61]. In our research, the neuroprotective effects of amorfrutin B disappeared in siRNA-transfected cells, i.e., after massive decreases in *Becn1* and *Atg7* mRNA expression (approx. 70 and 60%), suggesting that the mechanism of amorfrutin B-evoked neuroprotection involves an inhibition of autophagy; however, other mechanisms are not excluded. Previously, we demonstrated that post-treatment with amorfrutin B protected brain neurons against hypoxia/ischemia-induced damage in a PPARγ-dependent manner [24]. Interestingly, PPARγ functions appeared to be strongly suppressed in adipocyte-specific *Becn1* knockout mice [62], which supports the loss of neuroprotection in *Becn1* siRNA-transfected cells in our present study. Furthermore, treatment with the PPARγ agonist 15d-PGJ2 decreased autophagy-related protein expression and LC3 immunoreactivity in mice subjected to cerebral ischemia–reperfusion injury after MCAO [63]. Although post-treatment with amorfrutin B reduced the expression levels of *Becn1*, *Nup62*, and *Ambra1* during hypoxia, it stimulated *Atg5* and the protein levels of MAP1LC3B and AMBRA1 during ischemia, supporting the ambiguous role of autophagy in the development of brain pathologies. It is generally accepted that autophagy can cause neuroprotection by enhancing the clearance of misfolded protein aggregates or act in parallel with neurodegenerative processes, particularly during hemorrhage, kainic acid-induced excitotoxicity, and hypoxia [7]. Unfortunately, the methylation rates of autophagy-related genes (*Map1lc3b*, *Atg7*, *Becn1*) did not support the altered protein expression levels following exposure to amorfrutin B, thus suggesting other regulatory mechanisms. Indeed, amorfrutin B also affected miRNA expression, and many of the miRNAs were oppositely regulated by amorfrutin B and hypoxia/ischemia.

According to the study, amorfrutin B downregulated many hypoxia-upregulated miRNAs, namely *miR-106b-5p*, *miR-125b-5p*, *miR-143-3p*, *miR-144-3p*, *miR-145-5p*, *miR-16-5p*, *miR-17-5p*, *miR-181c-5p*, *miR-181d-5p*, *miR-183-5p*, *miR-195-5p*, *miR-20a-5p*, *miR-26b-5p*, *miR-29a-3p*, *miR-34a-5p*, *miR-365a-3p*, *miR-409-3p*, and *miR-7-5p*. Amorfrutin B also enhanced the expression of several miRNAs that were diminished during the course of hypoxia, such as *miR-146a-5p*, *miR-155-5p*, and *miR-1-3p*. In addition, amorfrutin B upregulated the expression of many ischemia-downregulated miRNAs, such as *let-7c-5p*, *miR-133a-3p*, *miR-134-5p*, *miR-16-5p*, *miR-222-3p*, *miR-31-5p*, *miR-34c-5p*, and *miR-98-5p*. Only *miR-205-5p*, which was increased by ischemia, was reduced by amorfrutin B. Since apoptosis and autophagy interfere with themselves, particularly with BECN1, BCL2, p53, and ATG5, the apoptosis-focused microarrays used in the present study also included autophagy-related miRNAs such as *miR-133a* and *miR-17-5p*. It has been shown that overexpression of *miR-133a* inhibits MPP^+^-induced autophagy (MAP1LC3A/MAP1LC3B, BECN1, NUP62) in a cellular model of Parkinson’s disease [64], and downregulation of *miR-17-5p* inhibits infarction-induced myocardial autophagy [65].

In our model of hypoxia, the majority of hypoxia-upregulated miRNAs have already been classified as markers of ischemic stroke and/or targets for novel treatments. These include *miR-106b-5p*, *miR-125b-5p*, *miR-143-3p*, *miR-145-5p*, *miR-17-5p*, *miR-181c-5p*, *miR-181d*, *miR-34a-5p*, *miR-365a-3p*, *miR-409-3p*, and *miR-7-5p*. Some other hypoxia-upregulated miRNAs, such as *miR-144-3p* and *miR-16-5p*, also appeared to be highly expressed during hemorrhagic stroke and myocardial infarction [66, 67]. Interestingly, the use of antagomir against *miR-106b-5p* or *miR-145-5p* reduced brain infarct volume and caused neuroprotection [68, 69], which is in line with the neuroprotective effect of amorfrutin B in our research, including the amorfrutin B-evoked downregulation of specific miRNAs. For the model of ischemia, decreased expression levels of miRNAs such as *let-7c-5p*, *miR-133a-3p*, *miR-222-3p*, *miR-34c-5p*, and *miR-98-5p* have previously been categorized as predictors of cerebral ischemia in humans as well as in animal and cellular models of ischemic stroke. Since overexpressing *let-7c-5p*, *miR-34c-5p*, and *miR-98-5p* decreased the infarction volume, attenuated neurological deficits, and inhibited apoptosis in MCAO mice [70–72], one may suggest that the amorfrutin B-evoked upregulation of the miRNAs observed in our study could be the mechanism of neuroprotection.

In summary, our study showed that a 6-h delayed post-treatment with amorfrutin B evoked strong neuroprotection against hypoxia and ischemia that was mediated by inhibiting apoptosis and autophagy and engaged regulatory mechanisms based on gene methylation and miRNA expression. Because amorfrutin B oppositely regulated hypoxia/ischemia through many miRNAs and interfered with various signaling pathways, targeting them could be a novel multifactorial therapeutic approach to improve stroke pharmacotherapy. The results strongly support the position of amorfrutin B among the most promising anti-stroke and wide-window therapeutics.

## Supplementary Information

Below is the link to the electronic supplementary material.

Supplementary file1 (DOCX 314 KB)

## Data Availability

The datasets generated during and/or analyzed during the current study are available from the corresponding author on reasonable request.

## Abbreviations

*Ambra1*/AMBRA1: Autophagy and beclin 1 regulator 1
ANOVA: Analysis of variance
*Atg5*: Autophagy-related protein 5
*Atg7*/ATG7: Autophagy-related protein 7
*Bax*/BAX: BCL2 associated X
BBB: Blood-brain barrier
*Bcl2*/BCL2: B-cell lymphoma 2
*Becn1*/BECN1: Beclin 1
CAT: Cationic amphiphilic tracer
Ct: Threshold cycle
DIV: Days in vitro
DMSO: Dimethyl sulfoxide
ELISA: Enzyme-linked immunosorbent assay
*Fas*/FAS: Cell surface death receptor
*Fasl*/FASL: FAS Ligand
GFAP: Filament glial fibrillary acidic protein
*Gsk3b*/GSK3β: Glycogen synthase kinase 3 beta
*Hprt1*: Hypoxanthine phosphoribosyltransferase 1
JNK: Jun N-terminal kinase
LDH: Lactate dehydrogenase
*Map1lc3a*: Microtubule associated protein 1 light chain 3 alpha
*Map1lc3b*/MAP1LC3B: Microtubule associated protein 1 light chain 3 Beta
MAPK: Mitogen-activated protein kinases
MCAO: Middle carotid artery occlusion
mTOR: Mammalian target of rapamycin
*Nup62*: Nucleoporin 62
PPARγ: Peroxisome proliferator-activated receptor gamma
qPCR: Quantitative polymerase chain reaction
ROI: Region of interest
rtPA: Recombinant tissue plasminogen activator
SPPARγM: Selective PPARγ modulator
TZDs: Thiazolidinediones
ULK1: Unc-51 like autophagy activating kinase 1
ULK2: Unc-51 like autophagy activating kinase 2
USP10: Ubiquitin specific peptidase 10
USP13: Ubiquitin specific peptidase 13
