# Supplementary material for: Amorfrutin B Protects Mouse Brain Neurons from Hypoxia/Ischemia by Inhibiting Apoptosis and Autophagy Processes Through Gene Methylation- and miRNA-Dependent Regulation
Source: Mol Neurobiol. 2022 Nov 3;60(2):576–95. doi: 10.1007/s12035-022-03087-9 (PMC9849175; doi:10.1007/s12035-022-03087-9)
Supplement: Supplementary file 1 — Supplementary file1 (DOCX 314 KB) [file 12035_2022_3087_MOESM1_ESM.docx]

**Supplementary materials**

1. **Results**
   1. **Administration of inhibitors specific for apoptosis- and autophagy-related pathways did not induce changes in LDH release in neocortical cultures under normoxic conditions**

As we previously described, treatment with Z-IETD-FMK, Z-LEHD-FMK, SP600125 and SB 203580 did not induce any changes in LDH activity in neuronal cell cultures [Rzemieniec et al., 2019]. Our current study showed that Z-DQMD-FMK, TDZD 8, temsirolimus, SBI-0206965, spautin-1 and MRT 68921 dihydrochloride also did not affect LDH levels, reaching 96%, 100%, 105%, 101%, 97% and 108%, respectively (Table S1a).

- 1. **Amorfrutin B did not affect mitochondrial membrane potential under normoxic conditions**

Compared to vehicle treatment, amorfrutin B did not affect the mitochondrial membrane potential. This parameter reached 100% of the control value (Table S1b).

- 1. **Effects of amorfrutin B treatment on apoptosis-related mRNA expression**

Compared to the control value, amorfrutin B did not induce any changes in mRNA expression levels, which amounted to 1.08-fold in the case of *Fas*, 1.15-fold in the case of *Fasl*, 0.95-fold in the case of *Bax*, 0.68-fold in the case of *Bcl2* and 1.05-fold in the case of *Gsk3b* (Table S1c).

- 1. **Effects of amorfrutin B treatment on autophagy-related mRNA expression**

Treatment with amorfrutin B did not affect the mRNA expression levels of *Becn1* (1.06-fold), *Map1lc3a* (1.14-fold) or *Ambra1* (1.07-fold). In turn, administration of the compound increased the expression levels of *Atg5*, *Atg7*, *Map1lc3b* and *Nup62* to 1.17-fold, 1.24-fold, 1.25-fold and 1.27-fold of the control, respectively (Table S1d).

- 1. **Effects of morfrutin B on apoptosis- and autophagy-related gene-specific methylation in neuronal cell cultures**

The methylation rates of *Bcl2* and *Atg7* reached 3% and 64%, respectively. The percentages of methylation of *Bax*, *Ambra1*, *Map1lc3b* and *Becn1* remained unchanged after amorfrutin B treatment in normoxic conditions (Table S1e).

- 1. **Effects of amorfrutin B treatment on the expression of apoptosis-focused** **miRNAs under normoxic conditions**

Under normoxic conditions, amorfrutin B treatment dysregulated 21 apoptosis-focused miRNAs. Our study showed that 8 miRNAs were downregulated (shown in green), including *miR-133a-3p*, *miR-133b*, *miR-145-5p*, *miR-200c-3p*, *miR-206*, *miR-29b-3p*, *miR-503-5p* and *miR-98-5p*. In turn, 13 miRNAs were upregulated (shown in red), including *miR-141-3p*, *miR-146a-5p*, *miR-15b-5p*, *miR-183-5p*, *miR-192-5p*, *miR-205-5p*, *miR-210-3p*, *miR-26b-5p*, *miR-29a-3p*, *miR-365a-3p*, *miR-409-3p*, *miR-451a* and *miR-466l-3p* (Fig. S1).


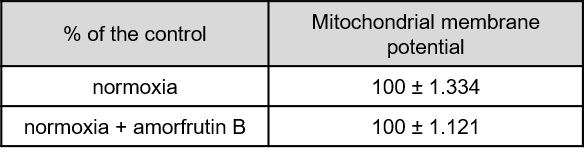

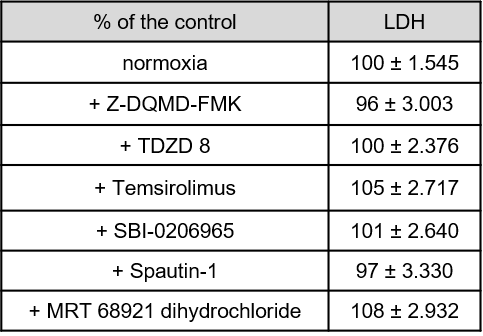

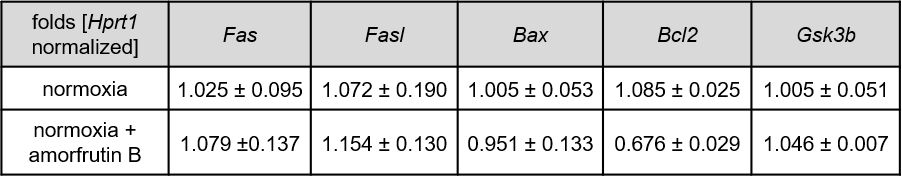

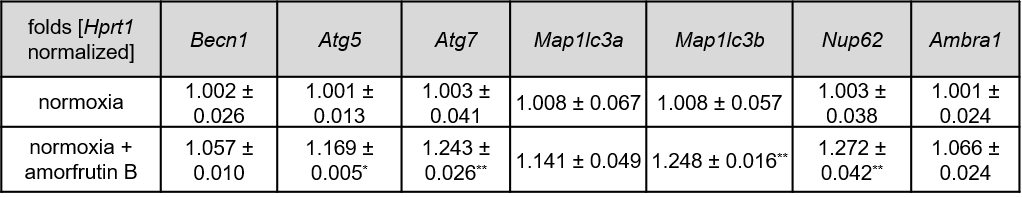

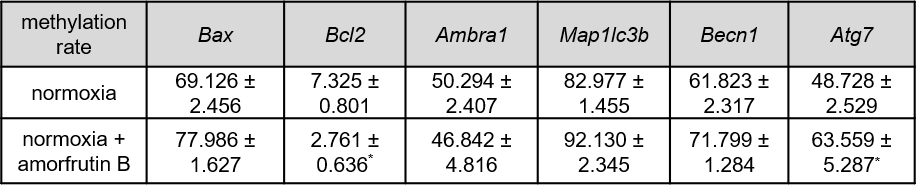


**a**

**b**

**c**

**d**

**e**

**Table S1** Effect of the apoptosis-/autophagy-related inhibitors (a) and amorfrutin B (b – e) post-treatment on LDH release, mitochondrial membrane potential, mRNA expression level and methylation rate under normoxic conditions. The results are presented as mean ± SEM. ^*^p < 0.05, ^**^p  <  0.01 compared to the control group.


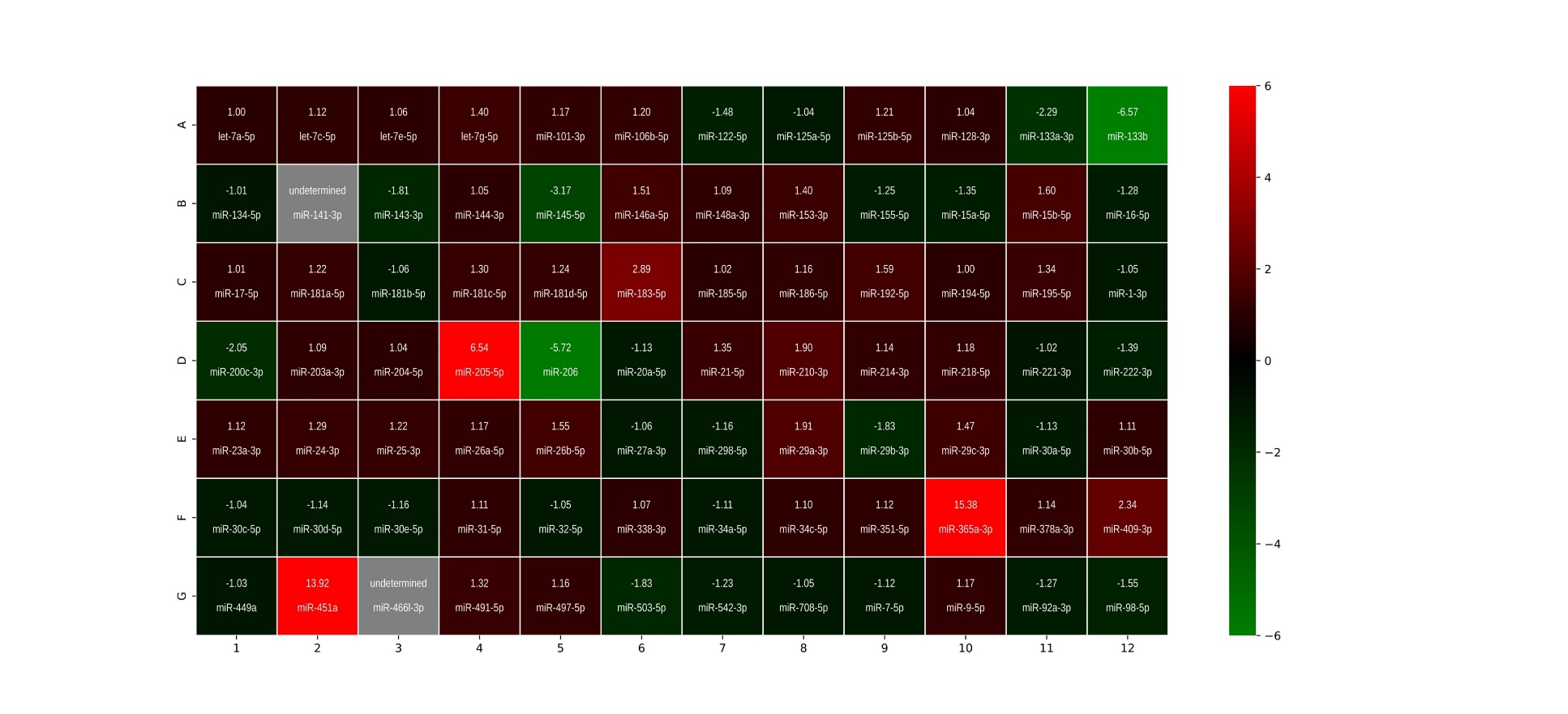


**Fig. S1** Changes in apoptosis-focused miRNA levels under normoxic conditions in response to amorfrutin B treatment. The results were determined by microarray analyses and are shown as heatmaps of 84 miRNAs. Downregulated miRNAs are shown in green, upregulated miRNAs are shown in red, and undetermined miRNAs are shown in gray.

1. **Genomic regions selected for methylation levels analysis**

***Bax***

Ensembl database

Gene: Bax ENSMUSG00000003873

Chromosome 7: 45103187-45113188 (promoter + promoter flank region)

***Bcl2***

Ensembl database

Gene: Bcl2 ENSMUSG00000057329

Chromosome 1: 106634987-106645494 (promoter + promoter flank region)

***Ambra1***

Ensembl database

Gene: Ambra1 ENSMUSG00000040506

Chromosome 2: 91559791-91563006 (promoter + promoter flank region)

***Map1lc3b***

Ensembl database

Gene: Map1lc3b ENSMUSG00000031812

Chromosome 8: 122302484-122321447 (promoter + promoter flank region)

***Atg7***

Ensembl database

Gene: Atg7 ENSMUSG00000030314

Chromosome 6: 114618927-114642745 (promoter + promoter flank region)

***Becn1***

Ensembl database

Gene: Becn1 ENSMUSG00000035086

Chromosome 11: 101190384-101194197 (promoter + promoter flank region)
